# Comparing the therapeutic efficacy of radiofrequency vs. Microwave ablation for non-functioning benign thyroid nodules

**DOI:** 10.1007/s40618-025-02734-x

**Published:** 2026-01-05

**Authors:** Marsida Teliti, Spyridon Chytiris, Rodolfo Fonte, Laura Croce, Linda Loretta Businaro, Silvia Anna Marchiselli Dell’ Innocenti, Francesca Coperchini, Flavia Magri, Mario Rotondi

**Affiliations:** 1 Department of Internal Medicine and Therapeutics, University of Pavia, 27100 Pavia, Italy; 2 Unit of Endocrinology and Metabolism, Laboratory for Endocrine Disruptors, Istituti Clinici Scientifici Maugeri IRCCS, 27100 Pavia, Italy

**Keywords:** Benign thyroid nodule, Radiofrequency ablation, Microwave ablation, Volume reduction rate, Thermal ablation

## Abstract

**Purpose:**

The aim of the study was to compare the efficacy, procedural features, and safety profile of radiofrequency ablation (RFA) versus microwave ablation (MWA) for the treatment of non-functioning benign thyroid nodules (BTNs).

**Methods:**

This retrospective, single-centre, cohort study included 203 nodules treated with RFA and 71 with MWA by a single experienced operator. Multivariate linear regression analysis was performed to identify independent predictors of treatment response, defined as 12-month volume reduction rate (VRR). Complication rates were also compared between RFA and MWA. A 1:1 case-control matching was performed based on baseline nodule volume and composition, yielding two matched cohorts of 66 patients each. VRRs after 6 and 12 months were compared between two groups.

**Results:**

In the overall population, similar VRRs were observed after 6 and 12 months. Significant differences in baseline nodule volume (24.35 ± 17.15 ml vs. 17.25 ± 15.22ml; *p* = 0.001) and in procedure times (519.96 ± 289.45s vs. 649.61 ± 321.05s; *p* = 0.003) were found for MWA and RFA, respectively. Multivariate analysis identified baseline volume (*p* < 0.001) and composition (*p* < 0.033) as significant and independent predictors of VRR. Both techniques showed comparable safety. In the matched cohorts, VRRs after 6-months were similar, while a higher VRR after 12-months was observed for MWA compared to RFA (64.70 ± 13.89% vs. 58.71 ± 16.81%; *p* = 0.028).

**Conclusion:**

RFA and MWA are both safe and effective for treating BTNs. In the overall population, VRRs were similar between RFA and MWA, with MWA requiring shorter procedure times. After matching for key predictors of therapeutic response, MWA demonstrated superior efficacy at 12-months.

## Introduction

Thyroid nodules are a common finding in clinical practice, with non-functioning, benign thyroid nodules (BTNs) being the most frequently detected [1]. Although most of these nodules are, and remain, rather asymptomatic, some may lead to compressive symptoms or cosmetic concerns, requiring therapeutic measures [2]. In recent years, minimally invasive techniques such as laser ablation (LA), radiofrequency ablation (RFA), microwave ablation (MWA), and high-intensity focused ultrasound (HIFU) have emerged as alternatives to surgery. Supported by a growing body of evidence on its efficacy and safety, thermal ablation (TA) is now recommended by current guidelines not only for patients ineligible for surgery but also as a first-line option in symptomatic or rapidly growing nodules [3–5].

All TA methods induce cytotoxic damage by heating tissue between 50 °C and 100 °C, but differ in how energy is delivered. LA was the first introduced, followed by RFA, while experience with MWA and HIFU is more limited [6]. According to the 2020 European Thyroid Association guidelines, LA and RFA are recommended as first-line therapies [5]. RFA has become the most widely used worldwide due to its extensive validation and availability, whereas MWA is still considered a second-line approach, though it may offer advantages such as higher intranodular temperatures, shorter procedure times, and more homogeneous ablation zones [5].

Several studies have aimed to compare the clinical efficacy and safety in the treatment of BTNs of both RFA versus MWA [7–17]. However, findings across studies remain heterogeneous, with some reports suggesting that RFA may lead to greater volume reduction at 6 months and beyond [7, 12, 16]. Also results from meta-analysis highlight considerable heterogeneity. While some meta-analyses confirmed that the two techniques offer comparable profiles in terms of efficacy and safety [18–20], others supported a better therapeutic outcome with RFA [21, 22]. Conversely, one meta-analysis found that MWA achieved higher mean VRRs compared to both RFA and LA, along with the lowest regrowth rate observed in the MWA group after three years [23].

This inconsistency likely reflects differences in patient populations, protocols, and operator expertise. Baseline factors such as nodule volume and composition, which strongly influence post-procedural outcomes [24–28], have not always been adequately controlled, contributing to variability across studies.

Given the growing use of TA, clarifying which technique may be preferred according to nodule characteristics is of clinical importance.

In light of these considerations, the present study was specifically designed to compare RFA and MWA in terms of therapeutic outcome, procedural aspects and patient safety, for treating solid or mixed non-functioning BTNs throughout a 12-month post-treatment follow-up. The fact that in our institution, both procedures are routinely performed by a single experienced operator, ensuring procedural consistency and minimizing operator-related variability would allow for a precise evaluation of the advantages and limitations of each technique. The primary endpoints of the study are: (1) comparison of the nodule volume reduction rate (VRR) between RFA and MWA, expressed as a percentage of baseline volume, and (2) comparative assessment of the safety profile of the two procedures.

## Materials and methods

This retrospective, single-centre, cohort study included 271 consecutive patients with non-functioning BTNs receiving RFA or MWA treatment at the Endocrinology and Metabolism Unit of the ICS Maugeri, Pavia between September 2021 and June 2024. The reasons for performing RFA or MWA included: (1) subjective compressive symptoms such as difficulty in swallowing or the feeling of local pressure and/or cosmetic concerns; (2) increase in the volume of the nodules over an ultrasound follow-up in the last years; (3) patient’s preference for thermal ablation rather than surgery. Inclusion criteria were: (1) patients diagnosed with benign thyroid nodules confirmed by at least one fine-needle aspiration cytology; (2) serum TSH, FT4, and FT3 levels within the normal range; (3) normal levels of serum calcitonin. Exclusion criteria were: (1) prior thyroid surgery or other thyroid interventions; (2) Incomplete follow-up data.

RFA was the first technique introduced in our center and was adopted in the initial phase. After the introduction of MWA in July 2023, both techniques were available, with MWA more frequently applied to larger nodules.

All patients referred for RFA or MWA treatment underwent a complete thyroid work-up, including a measurement of serum FT4, FT3, and TSH. A baseline US of the neck was performed by the same endocrinologist (S.C) who performed the procedures. The thyroid nodule’s location, volume, and characteristics were collected. Based on sonographic appearance, nodules were categorized as solid-predominantly solid when the solid component was ≥ 80% of the total volume, or as mixed when the cystic component was ≥ 20%. Nodules with a cystic portion exceeding 80% were excluded from the analysis. Thyroid nodule volume was calculated using the ellipsoid volume formula as follows: volume = 0.525 × length × width × depth.

To minimize bias related to multiple treatment sessions and to allow for an objective comparison of the clinical efficacy and safety of percutaneous RFA and MWA, only a single ablation session was performed per target nodule in both groups.

All patients gave written informed consent for the future use of their anonymized clinical and pathological data for research purposes, in accordance with applicable data protection and privacy regulations. The study was approved by the Ethics Committee of ICS Maugeri, Pavia (Protocol No. 2403 CE, approval date: March 31, 2020), and was conducted in compliance with the Declaration of Helsinki and relevant national ethical standards.

### Thermal ablation protocol

The procedures were performed in a sterile setting. Local anesthesia with Lidocaine 2% was administered at the skin puncture site and the perithyroidal space. No hydro dissection or anesthetic infusion was made in the peri- or under-capsular layer. All the procedures were performed by a single operator (S.C.) under ultrasound guidance. Energy settings, ablation duration, and post-procedure care were recorded. A dual sistem ablation generator, driving either a RF monopolar electrode or a MW mini-choked antenna through the same generator output port, was utilized (Amica Gen: Dual ablation system RF & MW-AGN-H.1.3, Hs Hospital Service S.p.A, Italy).

All RFA procedures were performed using a 18-gauge internally cooled electrode, 7–10 cm length with a 10 mm active tip. The starting level of energy delivered per second was 30 W. The energy output was progressively increased if no response was visible on the US within 60 s.

All MWA procedure were performed using a 17-gauge internally cooled electrode, 10 cm length. The level of energy delivered per second ranged from 20 W to 30 W.

At the end of the procedure, the patient applied mild compression of the treated thyroid lobe for 20 min. If necessary, an icepack was applied to relieve pain. After 2 h of post-procedural observation, a neck US was performed. Standard follow-up included outpatient visits with FT4, FT3, and TSH after 6 and 12 months. Nodule volumes were measured by the US after 6 and 12 months. Nodal volume reduction was expressed as volume reduction ratio, calculated as follows: VRR = ((initial volume − final volume)/(initial volume)) × 100.

### Statistical analysis

Statistical analysis was performed using the SPSS software version 30 (SPSS, Inc., Evanston, IL). Continuous variables are expressed as median (25th–75th percentiles)/mean (± SD). Qualitative data were expressed as frequencies. Comparison between groups was performed using the *χ* ^2^-test with Fisher’s correction for frequencies and the Student *t* test or Mann–Whitney *U* test for continuous variables. A multivariable linear regression model was designed including a mean VRR as a dependent variable and age, sex, nodule composition, nodule location, nodule baseline volume, and type of treatment (RFA or MWA) as covariates. This model was applied to the overall cohort. To minimize baseline imbalances and potential confounding, a 1:1 case-control matching was implemented. Patients treated with MWA were matched to those treated with radiofrequency RFA based on two key baseline variables: nodule composition and baseline volume. Matching was conducted with exact tolerance (0) for nodule composition (solid-predominantly solid vs. mixed), and a maximum tolerance of ± 1.5 mL for baseline nodule volume. All subsequent comparative analyses between MWA and RFA were conducted on this matched cohort. A *p* value < 0.05 was considered statistically significant.

## Results

### Overall population

A total of 203 patients underwent radiofrequency ablation (RFA) and 68 patients received microwave ablation (MWA), accounting for 203 and 71 treated nodules, respectively. Table 1 summarizes the baseline demographic, anatomical, and procedural characteristics of all patients undergoing thermal ablation, stratified by treatment modality (RFA vs. MWA). No significant difference was found in terms of mean age (RFA: 58.29 ± 12.95 years; MWA: 59.43 ± 14.88 years; *p* = 0.548), rates of male patients (13.8% for RFA vs. 11.8% for MWA; *p* = 0.670), rates of solid or predominantly solid nodules and mixed nodules (*p* = 0.121) as well as nodule location (*p* = 0.107) between the two groups.

**Table 1:** Baseline demographic, anatomical, and procedural characteristics of the overall population, stratified by treatment modality (RFA or MWA)

	All patients	RFA group	MWA group	*p* value
Patients, n	271	203	68	
Nodules, n	274	203	71	
Age, years	58.58 ± 13.45	58.29 ± 12.95	59.43 ± 14.88	0.548
Male (%)	36 (13.3%)	28 (13.8%)	8 (11.8%)	0.670
Nodule location (%)				0.107
Right	123 (44.9%)	87 (42.8%)	36 (50.7%)	
Isthmus	8 (2.9%)	4 (2.0%)	4 (5.6%)	
Left	143 (52.2%)	112 (55.2%)	31 (43.7%)	
Nodule composition (%)				0.121
Solid- predominantly solid	114 (41.6%)	90 (44.3%)	24 (33.8%)	
Mixed	160 (58.4%)	113 (55.7%)	47 (66.2%)	
Nodule baseline volume, ml	19.08 ± 16.03	17.25 ± 15.22	24.35 ± 17.15	**0.001**
Energy applied, J	16957.62 ± 12455.16	20365.37 ± 12485.77	6884.71 ± 4165.43	**< 0.001**
Energy per ml, J/ml	1285.44 ± 1222.00	1606.47 ± 1255.97	335.49 ± 170.04	**< 0.001**
Ablations, n	11.35 ± 6.9	8.75 ± 3.91	18.65 ± 8.20	**< 0.001**
Procedure time, sec	615.98 ± 317.76	649.61 ± 321.05	519.96 ± 289.45	**0.003**

Continuous variables are expressed as mean ± SD. Values in bold indicate statistically significant differences (*p* value < 0.05)

Of note, the baseline nodule volume was significantly larger in the MWA group compared to the RFA group (24.35 ± 17.15 ml vs. 17.25 ± 15.22 ml; *p* = 0.001).

From a procedural standpoint, RFA was associated with significantly greater energy delivery, both total (20365,37 ± 12485,77 J vs. 6884.71 ± 4165,43 J; *p* < 0.001) and per milliliter (1,606.47 ± 1,255.97 J/mL vs. 335.49 ± 170.04 J/mL; *p* < 0.001). Conversely, MWA required a higher number of ablations (18.65 ± 8.20 vs. 8.75 ± 3.91; *p* < 0.001) and was associated with a significantly shorter procedure time (519.96 ± 289.45 s vs. 649.61 ± 321.05 s; *p* = 0.003).

The therapeutic efficacy of both techniques was compared throughout the follow-up, showing similar efficacy. The mean VRR was 59.47 ± 16.22% for RFA and 58.10 ± 14.77% for MWA at 6 months (*p* = 0.530), and 62.01 ± 17.16% for RFA and 64.01 ± 14.27% for MWA, at 12 months (*p* = 0.379).

To evaluate the factors influencing treatment efficacy, a multivariate linear regression analysis was performed with mean VRR at 12 months as the dependent variable, and age, sex, nodule composition, nodule location, baseline nodule volume, and type of treatment (RFA or MWA) as covariates. As reported in Table 2, the analysis identified baseline nodule volume (B = −0.337; 95% CI: −0.460- 0.213; *p* < 0.001) and nodule composition (B = 4.103; 95% CI: 0.326–7.879; *p* = 0.033) as independent and significant predictors of VRR. In contrast, treatment modality, age, sex, and nodule location did not show a significant impact on volume reduction at 12 months.

**Table 2:** Multivariate linear regression analysis of predictors of nodule volume reduction rate (VRR) at 12 months in the overall population

Independent variables	Coefficent B	Lower limit 95% CI	Upper limit 95% CI	*p*-value
Age (years)	−0.048	−0.192	0.096	0.511
Male/Female	2.420	−3.097	7.937	0.389
Nodule location	−1.294	−4.669	2.081	0.451
Nodule composition (Solid-predominantly solid or Mixed)	4.103	0.326	7.879	**0.033**
Nodule baseline volume	−0.337	−0.460	−0.213	**< 0.001**
Treatment (RFA or MWA)	3.908	−0.414	8.229	0.076

RFA, Radiofrequency Ablation; MWA, Microwave Ablation. Values in bold indicate statistically significant differences (*p* value < 0.05)

### Matched‑cohort analysis: comparative VRR at 6 and 12 months in RFA vs. MWA

In order to minimize baseline imbalance between the RFA and MWA groups, and in light of the independent impact of baseline nodule volume and composition on 12-month VRR, a 1:1 case-control matching procedure was carried out. Each patient treated with MWA was matched to a patient treated with RFA based on the same nodule composition and a maximum allowable difference of ± 1.5 mL in baseline nodule volume. As shown in Table 3, the two matched cohorts showed overlapping baseline characteristics.

**Table 3:** Baseline demographic, anatomical, and procedural characteristics of matched patients undergoing radiofrequency ablation (RFA) and microwave ablation (MWA)

	RFA	MWA	*p* value
Patients, n	66	63	
Nodules, n	66	66	
Age, years	56.05 ± 13.67	58.22 ± 14.66	0.385
Male (%)	9 (13.6%)	8 (12.7%)	1.000
Nodule location (%)			0.463
Right	29 (44.0%)	35 (53.0%)	
Isthmus	2 (3.0%)	3 (4.6%)	
Left	35 (53.0%)	28 (42.4%)	
Nodule composition (%)			1.000
Solid- predominantly solid	21 (31.8%)	21 (31.8%)	
Mixed	45 (68,2%)	45 (68.2%)	
Nodule baseline volume, ml	21.42 ± 13.35	21.55 ± 13.43	0.957
Energy applied, J	19995.85 ± 11938.95	6557.19 ± 3986.90	**< 0.001**
Energy per ml, J/ml	1071.96 ± 653.79	342.80 ± 172.66	**< 0.001**
Ablations, n	8.14 ± 3.71	17.91 ± 7.50	**< 0.001**
Procedure time, sec	634.02 ± 332.26	482.80 ± 247.61	**0.004**

Continuous variables are expressed as mean ± SD. Values in bold indicate statistically significant differences (*p* value < 0.05)

**Fig. 1:**
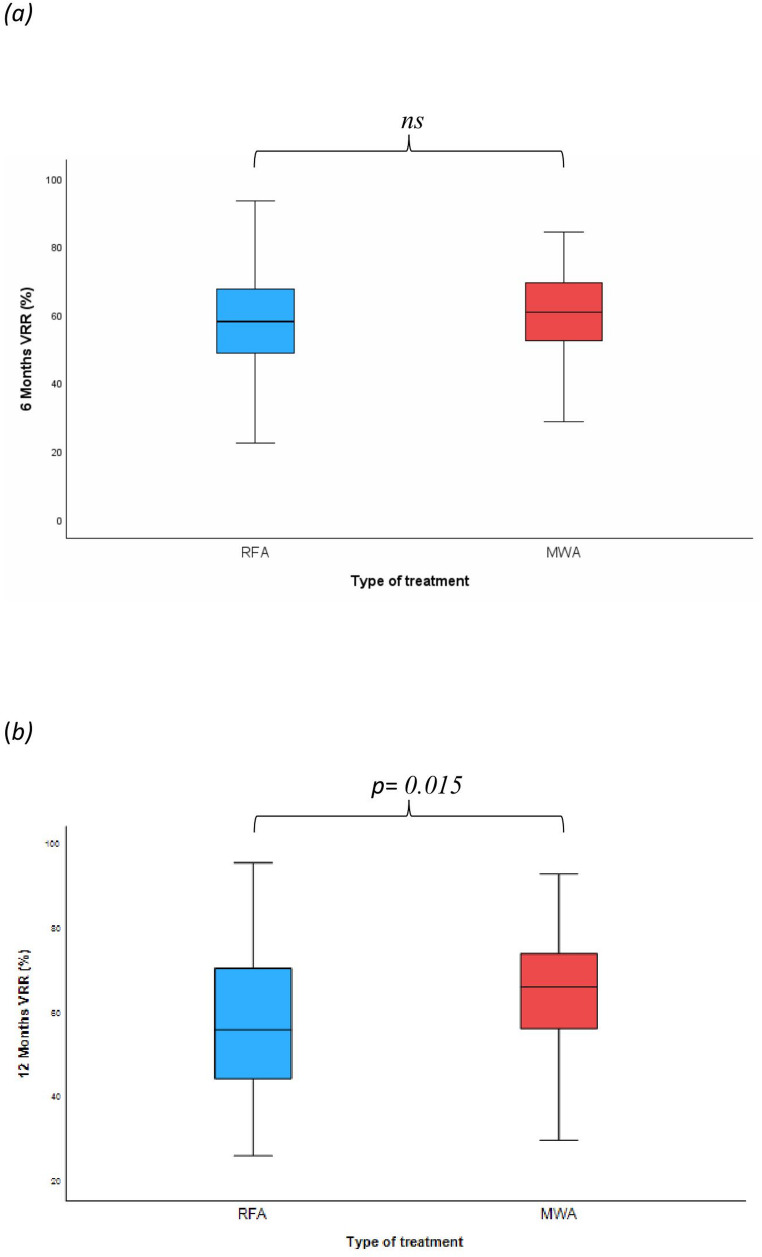
Distribution of volume reduction rate (VRR) values for RFA and MWA treatments at 6 months (a) and 12 months (b) in the matched cohort (a) After 6 months, the median VRR was comparable between the two groups: 58.14% (range 22.28–93.30) for RFA and 60.19% (range 1.21–84.34) for MWA (*p* = 0.431, Mann–Whitney U test) (b) After 12 months, the MWA group showed a significantly higher median VRR of 66.35% (range 22.37–93.18) compared to 56.18% (range 26.33–95.81) in the RFA group (*p* = 0.015, Mann–Whitney U test) Data are expressed as medians, with 25th and 75th percentiles represented by boxes and 5th and 95th percentiles by whiskers

Thereafter, the VRR after 6 and 12 months were compared between the two treatment groups. Similar mean VRR (57.77 ± 14.59% vs. 58.75 ± 14.74%, for RFA and MWA, respectively; *p* = 0.702;) were observed after 6 months. At difference, comparison of the mean VRR after 12 months highlighted a significant difference in the mean VRR which was higher for MWA (64.70 ± 13.89%) as compared to RFA (58.71 ± 16.8 1%; *p* = 0.028, Student *t* test). Figure 1 shows the distribution of VRR values for RFA and MWA, including medians and percentile ranges.

### Peri- and post-procedural complications

Peri- and post-procedural complications were uncommon in both groups and were generally mild and self-limiting. The distribution of adverse events is summarized in Table 4. Hematoma occurring during the procedure was observed in 3 patients in the RFA group (1.48%) and in 2 patients in the MWA group (2,81%). Transient dysphonia, likely related to thermal injury or nerve irritation, was reported in one RFA-treated patient (0.49%) and resolved spontaneously within a few weeks. Severe pain during the procedure was reported in 1 patient in each group (0.49% in RFA, 1.4% in MWA), requiring temporary interruption of the ablation. In the MWA group, two additional post-procedural complications were recorded: one case of granuloma formation at the treatment site (1.4%) and one case of localized inflammation involving the sternocleidomastoid muscle (1.4%). Both conditions were managed conservatively with complete resolution. No major complications were reported in either group. A comparative analysis between RFA and MWA groups showed no statistically significant differences in the overall complication rate (5/203 [2.46%] for RFA vs. 5/71 [7.04%] for MWA; *p* = 0.132). Similarly, no individual complication showed a statistically significant difference in incidence between the two groups.

**Table 4:** Peri- and Post-Procedural complications observed after radiofrequency ablation (RFA) and microwave ablation (MWA)

Complications	RFA (*n* = 203)	MWA (*n* = 71)
Hematoma	3 (1.48%)	2 (2.81%)
Transient dysphonia	1 (0.49%)	0 (0%)
Severe pain during the procedure	1 (0.49%)	1 (1.4%)
Granuloma	0 (0%)	1 (1.4%)
Sternocleidomastoid muscle inflammation	0 (0%)	1 (1.4%)

## Discussion

The present study was specifically aimed at comparing RFA and MWA for the treatment of non-functioning BTNs, in terms of treatment efficacy, procedural aspects, and safety. The results obtained in the overall study population showed that RFA and MWA lead to similar VRR after 6 and 12 months. The results of the multivariate analysis identified both baseline nodule volume and composition as significant and independent predictors of treatment response. Thus, the here reported similar efficacy between RFA and MWA should take into account that baseline nodule volumes were significantly larger in the MWA group.

Previous studies on the topic, consistently reported that smaller and mixed nodules tend to respond more favorably to thermal ablation, being larger nodules generally associated with reduced responsiveness to thermal ablation regardless of the technique used [24–26, 28]. This could indirectly suggest that the similar results achieved by MWA and RFA in a population with significant differences as to the baseline nodules volume might indicate that MWA would offer greater versatility in treating nodules of varying sizes.

Moreover, MWA required a significantly shorter procedure times, even if treating larger nodules, which is of not-negligible relevance, as minimizing treatment duration may improve comfort and tolerability. This relatively greater procedural efficiency, is likely due to the ability of MWA to deliver higher temperatures, reduce the impact of tissue impedance, and achieve more uniform thermal distribution. Taking together the above and in order to avoid potential confounding effect of baseline differences in treated nodules, that could impair a precise comparison of the two TA techniques, a 1:1 case-control matching based on nodule volume and composition was performed. The comparison of the VRR after 12 months in the matched cohort, showed that MWA was associated with a significantly higher VRR, confirming the trend observed in the unmatched analysis, suggesting a more sustained and progressive effect over time with MWA. On the other hand, the similar findings between the two techniques after 6 months indicate that early responses may not fully capture the long-term dynamics of tissue remodeling following MWA.

The present results should be considered in light of two previous randomized trials directly comparing RFA and MWA. Javadov et al. reported similar outcomes after 6 months in solid benign nodules, although details on operator experience and procedural standardization were lacking [14]. Chen et al. confirmed comparable efficacy at 2 years, but the small mean baseline volume (9.1 ± 9.0 mL) and the use of multiple operators may limit the generalizability of their findings [9]. Most other studies are retrospective and affected by baseline imbalances [12, 15]. Finally, the exclusion of cystic or predominantly cystic nodules, not consistently adopted across studies, helps reducing bias related to cyst aspiration, which may otherwise overestimate VRR.

The safety analysis confirmed that both RFA and MWA techniques are generally well tolerated, with no major complications and only minor, self-limiting adverse events. Although the rate of minor complications was slightly higher in the MWA group, this difference did not reach statistical significance.

The fact that in the present study, all procedures were performed by a single experienced operator has likely minimized the inter-operator variability, a factor that has not been taken into account by previous comparative studies. Thus, it is possible that baseline imbalances (nodule’s volume and composition), heterogeneity in procedural framework (e.g. differences in generators, electrode configurations, applied power, and ablation durations) and differences in the operator’s skill may explain the heterogeneity of previously published results leading to a lack of consensus regarding the most effective technique.

Indeed, only two studies clearly state that both RFA and MWA were performed by the same operator, a key condition to reduce inter-operator bias [12, 16]. Interestingly, a recent meta-analysis reported superior outcomes with RFA compared to MWA when performed by less experienced operators (< 10 years) [22], suggesting a gentler learning curve for RFA [29–33].

A major limitation of the present study stems from the lack of long-term follow-up beyond 12 months precludes evaluation of late regrowth rates. Furthermore, although the use of the same dual-modality generator represents a strength in terms of standardization, it may limit the generalizability of our findings to centers using different devices. Finally, patient-reported outcomes were not included, as the study was primarily designed to compare objective efficacy measures; however, their integration in future research may offer additional complementary insights.

In conclusion, the results of the present study demonstrate that both RFA and MWA represent effective and safe options for the treatment of non-functioning BTNs. Our findings suggest that MWA achieves comparable efficacy to RFA even in the presence of significantly larger nodules and offers the added benefit of shorter procedure times. The use of a matched cohort undergoing either RFA or MWA and the fact that a single skilled operator performed both techniques allowed for a more rigorous comparison by controlling for major predictors of treatment response (baseline nodule volume and composition, and operator performance). The comparison of the 12 months VRR between RFA and MWA, after matching for key predictors of therapeutic response, showed a greater therapeutic efficacy for MWA. Overall, these findings indicate that MWA may be particularly suitable for patients with large, predominantly solid nodules and may be especially advantageous in elderly or comorbid individuals, for whom shorter procedure time improves comfort and tolerability. Future prospective studies with long-term follow-up and evaluation of patient-centered outcomes are warranted to further define the optimal use of thermal ablation techniques in clinical practice.

## Data Availability

The datasets generated during and/or analysed during the current study are available from the corresponding author on reasonable request.
